# Navigational strategies underlying phototaxis in larval zebrafish

**DOI:** 10.3389/fnsys.2014.00039

**Published:** 2014-03-25

**Authors:** Xiuye Chen, Florian Engert

**Affiliations:** Department of Molecular and Cellular Biology, Harvard UniversityCambridge, MA, USA

**Keywords:** phototaxis, zebrafish, navigation, modeling, behavior

## Abstract

Understanding how the brain transforms sensory input into complex behavior is a fundamental question in systems neuroscience. Using larval zebrafish, we study the temporal component of phototaxis, which is defined as orientation decisions based on comparisons of light intensity at successive moments in time. We developed a novel “Virtual Circle” assay where whole-field illumination is abruptly turned off when the fish swims out of a virtually defined circular border, and turned on again when it returns into the circle. The animal receives no direct spatial cues and experiences only whole-field temporal light changes. Remarkably, the fish spends most of its time within the invisible virtual border. Behavioral analyses of swim bouts in relation to light transitions were used to develop four discrete temporal algorithms that transform the binary visual input (uniform light/uniform darkness) into the observed spatial behavior. In these algorithms, the turning angle is dependent on the behavioral history immediately preceding individual turning events. Computer simulations show that the algorithms recapture most of the swim statistics of real fish. We discovered that turning properties in larval zebrafish are distinctly modulated by temporal step functions in light intensity in combination with the specific motor history preceding these turns. Several aspects of the behavior suggest memory usage of up to 10 swim bouts (~10 sec). Thus, we show that a complex behavior like spatial navigation can emerge from a small number of relatively simple behavioral algorithms.

## Introduction

Quantitative insights into how the brain transforms sensory input to motor output are essential to understanding the neuronal basis of complex behavior. Ideally, a well-designed behavioral assay in combination with careful analysis can reveal a sequence of relatively simple algorithms that underlie these complex transformations (Marr, 1982). These algorithms can then serve as testable hypotheses for studying the physiological properties of the underlying neuronal circuitry.

An interesting and important class of behaviors that is well-suited for such algorithmic analysis is taxis—an innate behavioral response to a directional stimulus or gradient of stimulus intensity. Examples of such stimuli and their respective taxes are light (phototaxis) (Sawin et al., 1994), chemicals (chemotaxis) (Louis et al., 2008), temperature (thermotaxis) (Mori and Ohshima, 1995), and gravity (geotaxis) (Toma et al., 2002). The movement may be directed toward the stimulus (positive taxis) or away from it (negative taxis).

In order to achieve taxis behavior, an organism needs to compare samples of sensory information to inform its directional movement, but this comparison can be made in space or in time, and therefore, two general types of strategies for taxis behavior can be distinguished. Spatial strategies involve instantaneous comparisons between stimuli intensities at different points in space, while temporal strategies compare stimulus intensity at successive moments in time. Fraenkel and Gunn (1961) term the spatial and temporal strategies “tropotaxis” and “klinotaxis,” respectively. A classic example of temporal taxis is the “biased random walk” strategy in bacteria chemotaxis (Segall et al., 1986). An *E. coli* bacterium compares the chemical concentration at different times, and when it is moving away from an attractant like a food source, the tumbling frequency increases. Phototaxis, on the other hand, provides an intuitive example of a spatial strategy. Whenever an image is formed, light intensities at different points of the visual field can be compared and this can directly influence the animal's direction of travel. In comparison, temporal strategies for phototaxis are largely understudied, with a few exceptions such as the negative phototaxis of the blowfly larva *Calliphora* (Fraenkel and Gunn, 1961) and dark photokineses in blind fish by means of deep brain photoreceptors (Fernandes et al., 2012). However, studies of temporally based decisions can be highly informative and necessarily include certain forms of memory, since each sample of sensory input needs to be compared to at least one previous set. An interesting question to ask is whether a vertebrate with binocular vision, such as the larval zebrafish, also employs temporal strategies in phototactic behavior. Such behaviors might be observed in natural environments when an animal enters a shadow, where the sudden darkening of the surroundings represents a temporal decrease in luminosity.

Larval zebrafish demonstrate positive phototaxis, that is, they are attracted by light and are averse to darkness (Brockerhoff et al., 1995; Orger and Baier, 2005). As demonstrated by Burgess et al. (2010), larval zebrafish respond to localized illumination by first turning toward the light source and then swimming forward. Another study showed that when a larval zebrafish is presented a left/right illumination contrast (black/white with sharp border at midline) that is stabilized relative to the fish's orientation, it turns robustly toward the white side (Huang et al., 2013). This shows that a purely spatial difference in luminosity is sufficient to elicit phototactic behavior in larval zebrafish, but leaves unresolved the question whether purely temporal changes in luminosity also contribute to phototactic behavior.

In order to develop a comprehensive understanding of phototaxis, we isolated its purely temporal component by presenting only spatially uniform stimuli—light and dark. To that end, we developed a novel “Virtual Circle” assay in which a virtual circular border (invisible to the fish) is defined within a uniformly illuminated arena. The lights are turned off when the fish crosses the border (leaving the circle), and are turned on again when the fish returns to within the circle. Despite the fact that the fish has no direct spatial information about the location of the virtual border, we find that its trajectory is remarkably well contained within the interior of the circle (or other geometric shapes as implemented in variants of this assay).

Since larval zebrafish swim in discrete bouts, this behavior can be modeled using discrete algorithms that transform the binary visual input (uniform light/uniform darkness) into specific swim properties. Analysis of the fish trajectories reveals four algorithms that modulate the swim turns (bouts) based on recent sensory and motor history: (1) the turning-angle magnitude is modulated by a light transition; (2) the (left/right) direction of turns following a light switch depends on the direction of the previous turn. Computer simulations show that these first two algorithms are sufficient to ensure that a fish spends most of its time within the virtual border. The latter two algorithms suggest usage of memory of up to 10 bouts (~10 sec); (3) the cumulative angle during a dark interval modulates the turning direction of the subsequent bouts in light, and (4) the probability of the fish changing the turning direction after entering the dark depends inversely on the duration of the preceding light interval. Algorithms (3) and (4) explain more complex properties of fish behavior like their general affinity to the virtual border and their ability to consistently turn in the direction that most quickly returns them to the virtual circle. These four algorithms inform us about how the larval zebrafish brain transforms purely temporal stimuli into a spatially well-defined behavior. In summary, this study characterizes temporal phototaxis in a vertebrate, and shows that this complex behavioral trait, which might appear to require an internal representation of space, can actually emerge from a small number of relatively simple computational rules.

## Materials and methods

### Fish and behavioral setup

**Figure 1:**
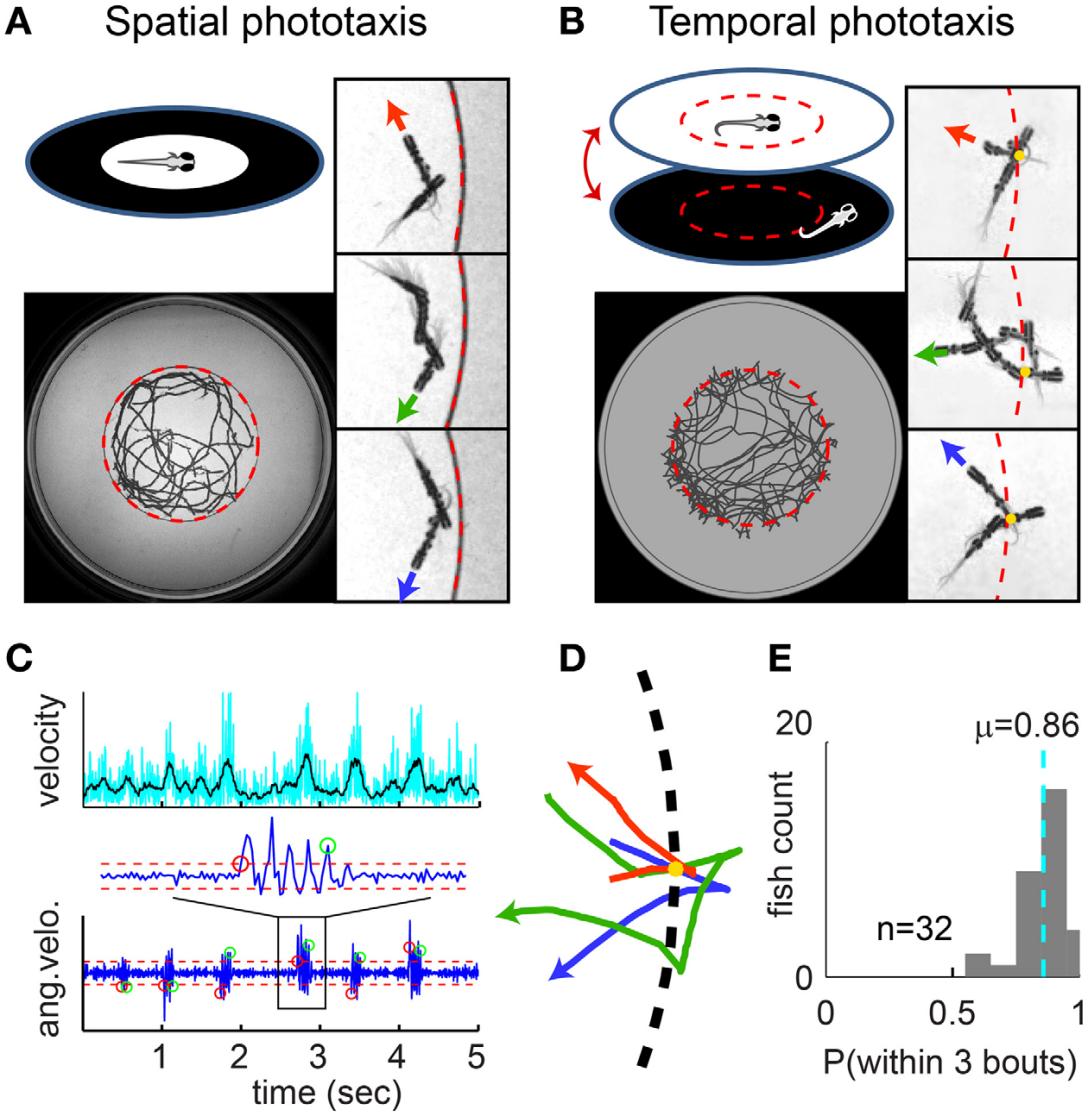
**Spatial vs. temporal phototaxis. (A)** Larval zebrafish prefer light over darkness in the spatial comparison assay. Upper left: a diffusive (scattering) white screen surrounded by an opaque black ring is placed beneath the arena (a transparent dish) and illuminated from below. Lower left: the full trajectory of a fish over a session of 8 min. Right panels: close-up views of trajectory segments close to the border. Three example segments are shown (rotated into the same orientation), with swim direction indicated by the red, green, and blue arrowheads; circular border indicated by the dashed red line. Note that the fish does not cross the border. **(B)** Temporal comparison assay, i.e., the Virtual Circle (VC) assay. The uniform illumination is turned off when the fish exits the virtual circle (dashed red circle, invisible to fish), and turned on again when the fish returns. Close-up view as in **(A)**, additionally with yellow dots marking the point where the fish exits the virtual circle. **(C)** Larval zebrafish swim in distinct bouts. Upper panel: velocity of the fish over time. Lower panel: bouts are determined by thresholding the angular velocity (i.e., per-frame change of heading angle); red/green circles mark the start/end of bouts, dashed red lines mark the thresholds. **(D)** Trajectory segments close to the virtual border are extracted from the VC assay, with the point where fish exits the border aligned to the yellow dot. Three example segments are shown in red, green and blue, with swim direction indicated by arrowhead. **(E)** The probability of each fish returning to light within 3 bouts of exiting the virtual circle, summarized as a histogram for all 32 fish. Dashed cyan line indicates population mean.

Wild-type zebrafish (*Danio rerio*, WIK strain) larvae 5–7 days-post-fertilization are used for the free-swimming experiment. Experiments are conducted on single fish during daytime hours. The entire experimental setting (not including the computer) is enclosed in a light-tight rig. The circular arena is made of a standard (transparent) petri dish 100 mm in diameter, with the side walls taped black (to reduce thigmotaxis, i.e., the preference for walls). White light from a Dell DLP projector is projected from below onto a diffusive screen placed directly under the dish, although for the original Virtual Circle experiments a white LED which provides even illumination was used. The screen and dish are illuminated with infrared from below. For Figure 1A and Figure S1C, images were captured with an infrared-sensitive CMOS camera (Mikrotron, MC1362) at 30 Hz, and the minimum intensity projections of fish trajectories are shown (on smoothened background image). For all other experiments, we used a different infrared-sensitive high-speed camera (AVT Pike F-032) that captures the motion of the fish at 208 frames per second. A custom program written in C# was used for all behavioral experiments. Online tracking of the location and heading direction of the fish was employed for both the administration of closed-loop experiments and data recording. For the playback experiments, online bout-determination uses the same method as in data analysis as shown in Figure 1C (thresholding the change of heading direction).

Fish were reared on a 14/10 h light/dark cycle at 28°C. Animal handling and experimental procedures were approved by the Harvard University Standing Committee on the Use of Animals in Research and Training (Cambridge, MA).

### Behavioral assays

#### “virtual circle”

The virtual border is defined as a circle of half the radius of the dish and concentric with the dish; it is only implemented in the programming code and is invisible to the fish. The light is only on when the location of the fish (determined by automatic online tracking) is within the borders. (Light on: uniform white projection onto a circular area that is larger than the arena; Light off: instead of projecting “black” from the projector, the LED's in the projector are turned off, so the arena is in complete darkness). Before a session start, fish are transferred to the arena and are allowed to adapt for 10 min. A fish at the edge (side wall) of the dish may spend a significant amount of time along the edge (thigmotaxis); therefore, when the fish swims out of an “exit line” very close to the edge (defined in the computer and invisible to the fish), the lights are turned on, and the experiment would be paused. The experiment resumes again when the fish swims back to the center (within an “entry line” that is defined within the virtual border). Trajectory during pauses are not analyzed. Each fish is tested for a session of 15 or 30 min in length including pausing time (*n* = 32 fish).

#### “playback” dark flashes

The duration of the intervals in light, and intervals in dark, respectively, are pooled from all fish in the VC assay into 2 corresponding probability distributions. For the Playback experiments, we draw from these two distributions alternatingly, to produce a sequence of Dark Flashes alternating with light intervals to present to naïve fish. To mimic the feature that in the VC assay, light switches only occur when the fish is moving, we also perform online bout-determination in the Playback experiments, and the light switches are only triggered when the fish is making a bout. More specifically, we extracted from the VC data that on average, a light switch is triggered 50 ms after a bout-start is determined online, so we subtract 50 ms from every interval in the sequence produced for the Playback experiments, and during the experiment, the light switches are triggered 50 ms after a bout-start is determined. Effectively, the Playback experiment serves as a “yoke” control to the VC assay; collectively these fish receive the equivalent visual input as the fish in the VC assay (same duration/frequency of Dark Flashes but shuffled).

Similar to the VC assay, when fish reach the edge of the arena, the experiment is paused, and we use a visual projection to encourage them to return to the center of the arena: a white circle (on black background) is projected to the center of the arena to attract the fish by phototaxis. This phototaxis attraction is not necessary for the VC assay as they rarely come to the edges, but in the Playback experiment it happens so often that without this attraction, the average interval between Dark Flashes may be too long to compare with the VC assay.

### Data analysis

**Figure 2:**
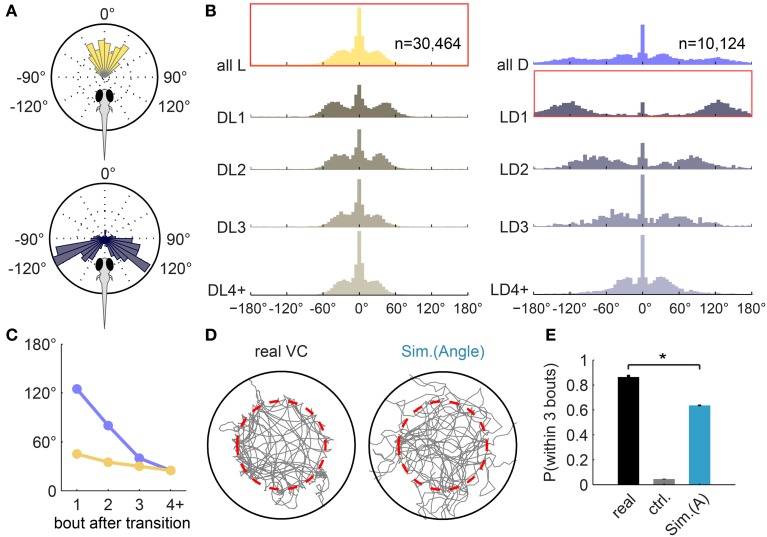
**Turning-angle distributions in the light, in the dark, and at transitions. (A)** Turning-angle distributions from an example fish. Upper panel: all turns in light. Lower panel: the first turn after the Light-to-Dark transition (LD1) is usually a large angle turn. **(B)** Turning-angle distributions from all 32 fish, for bouts in light (left panel) and in dark (right panel). The top two histograms summarize all turns made in light (left) and in dark (right), respectively. “DL*n*”: the *n*-th turn after a Dark-to-Light transition. DL4+: all subsequent turns in the dark. “LD”: respective turns in response to a Light-to-Dark transition. Histograms framed in red correspond to the single-fish polar plots in **(A)**. **(C)** Mean turning angle—after excluding center peaks—of the *n*-th turn after transitions, extracted from **(B)**. **(D)** Example trajectories of real and simulated sessions (using Algorithm I [Angle], i.e., turning-angle distributions in **B**) for the Virtual Circle assay. Fish trajectory is shown in gray; dashed red circle marks the virtual border. Traces after the fish reached the edge of the arena and before it returned to within the virtual borders are not shown (see Materials and Methods). **(E)** The average probability of returning to light within 3 bouts of exiting the virtual circle (similar to Figure 1E), compared between real fish (*n* = 32), a control simulation (“ctrl,” *n* = 100) that implements a generic turning-angle distribution pooled from all turns, and the simulation (*n* = 100). [Mean ± s.e.m, ^*^p < 0.001 (paired *t*-test) for all pairs].

All data analysis was performed with custom code written in MATLAB, Mathworks (code available in Supplementary Material). For bout determination (Figure 1C), the threshold is determined empirically. For the turning angle distributions in Figure 2B, all histograms are normalized to have the same area—regardless of sample size for the different categories. The sample sizes for the categories “all L,” “DL1,” “DL2,” “DL3,” “DL4+” are 30464, 3386, 3234, 2878, 20974, respectively, and for “all D,” “LD1,” “LD2,” LD3,” “LD4+” are 10124, 3386, 1292, 658, 4788, respectively.

**Figure 3:**
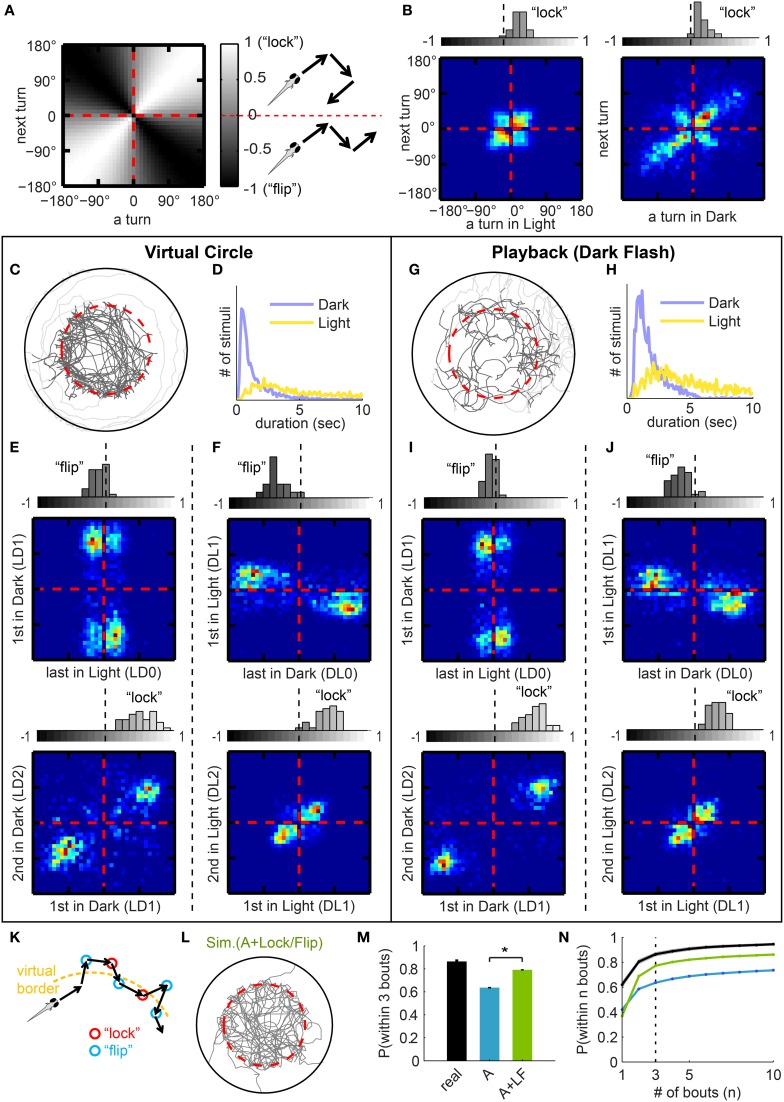
**Turning modulation around light transitions. (A)** Value map of “lock-index” for correlation matrices **(B,E,F,I,J)**. For a pair of consecutive turns, “locked” turns (2 turns in the same direction) populate the white diagonal (correlation), and “flipped” turns (2 turns in opposite directions) the black diagonal (anti-correlation). The lock-index ranges from −1 to 1, −1 being “flipped” (with identical turning magnitudes) and 1 being “locked.” **(B)** Correlation matrices for consecutive turns, data pooled from all 32 fish. Left panel: all pairs of consecutive turns in light. Most are small angle turns, and fish have a small bias for “lock.” Right panel: all pairs of consecutive turns in dark. Most large angle turns are “locked” in the same direction. Above each matrix: corresponding histograms of average lock-indices for each fish, calculated with **(A)**. **(C)** Sample trajectory of Virtual Circle experiment. Traces after the fish reached the edge of the arena and before it returned to within the virtual borders are colored in light gray. **(D)** Distribution of duration of stimuli (Dark intervals or Light intervals, respectively), pooled from all fish. **(E,F)** Correlation matrices with corresponding histograms similar to **(B)**, but for different specific categories of turns. **(E)** For 2 groups of turns around Light-to-Dark transitions, as indicated on axes. Note that the pair of turns surrounding the transition is “flipped,” and the pair immediately afterward is “locked.” **(F)** Similar to **(E)**, but for turns around Dark-to-Light transitions. **(G,H)** Playback experiment: visual stimuli from VC assay (Dark Flashes) played-back to naïve fish (see text). **(G)** Sample trajectory of the playback experiment. **(H)** Actual distribution of stimuli duration of the playback experiment, from 27 fish. **(I,J)** Compare to **(E,F)**, but from the playback experiment. The similarity to **(E,F)** indicates that the lock-flip tendencies do not depend on a specific geometry of the virtual border. (**K)** Illustration of a model in which both “lock” and “flip” turns tend to lead the fish back toward the virtual border. **(L)** Example session from a simulation (displayed as in Figure 2D) that implements both Algorithm I [Angle] (from Figure 2B) and Algorithm II [Lock/Flip] (from **B,E,F**). (**M)** The average probability of returning to light within 3 bouts (expansion of Figure 2E). Compared between real fish (*n* = 32) and the two simulations (*n* = 100 each). Simulations are labeled by the algorithms implemented: “A” = Algorithm I [Angle], “LF” = Algorithm II [Lock/Flip]. [Mean ± s.e.m, ^*^p < 0.001 (paired *t*-test) for all pairs.]. **(N)** Probability that a fish returns to light within *n* bouts, mean ± s.e.m plotted as a function of *n*, color-coded as in **(M)**.

The correlation matrices in Figure 3 are normalized to represent each fish equally. The lock-index is calculated similar to a dimensionless correlation value. For a single pair of consecutive turns, lock-index = *x* · *y* ·2/(*x*^2^ + *y*^2^), where *x*, *y* are the turning-angles of the 2 turns, respectively. The value map shows the lock-index of all combinations of two binned turning-angles in grayscale in a 2D array. The lock-index reaches its minimum and maximum (−1 and 1) when the amplitudes of the 2 turning angles are identical. For Figure S4A, the calculation of the density at the border is based on all trajectories but excluding the segments where the fish gets “lost,” because if the fish leaves the virtual circle frequently (as in the “basic” simulation), the density at the border is naturally diluted compared to a fish that is tightly confined within the virtual circle. For this calculation, trajectory segments where the fish doesn't return to the virtual border within 3 bouts are excluded, for the data from real fish as well as the 3 simulations.

### Simulation

All simulations are custom written in MATLAB (source code available in Supplementary Material). Since larval zebrafish swim in discrete bouts, we constructed a discrete model that simulates the trajectory of a fish bout by bout. The dimensions of the simulated arena and virtual circle are designed to match the real VC assay. The displacement for a bout are approximately constant for real fish, and is hold constant in the simulation. The simulated fish starts in the center of the simulated arena (within the virtual border), and a heading-direction is randomly assigned. Then for each bout, light on/off is determined based on the updated position (in relationship to the virtual border in the simulation); the turning angle of the next bout is determined by algorithms as described below. If the fish reaches the outer bound of the arena, the simulation “restarts” from a random location within the virtual border. For each set of parameters, the simulation is performed for *n* = 100 sessions.

Mainly, 4 progressive versions of simulations have been performed: the “basic,” “lock-flip,” “bounce,” and “efficient” version, respectively. Except for the “basic” version, each simulation is based on the previous version, only with new algorithms added.

The “basic” version, implementing Algorithm I, only uses the gray-shaded turning angle distributions from Figure 2B (without any history-dependent modulations). For each bout, first the program determines which category this bout belongs to (e.g., “first turn after light-on,” or “beyond 4th turn after light-off”), then draws an angle from the corresponding probability distribution (corresponding normalized histogram).

The “lock/flip” version, implementing Algorithms I+II, incorporates the “lock/flip” patterns from Figures 3B,E,F. Given a bout in the “lock/flip” simulation, the signed turning angle of the next bout is obtained by drawing randomly from the marginal probability distribution (given the turning angle of the current bout) from the probability matrices obtained by normalizing Figures 3B,E,F.

For the “bounce” version, implementing Algorithm I+II+III, the cumulative angle turned in a dark interval is calculated after the fish re-enters the circle, and as the trajectory is simulated bout by bout in the light, the cumulative angle turned in light is kept updated, and the direction of the next turn is adjusted to approach the desired angle—the cumulative angle turned in dark but with reverse sign. Only the direction and not the magnitude of the turns are adjusted, so this does not affect the turning angle distributions; and only the turns at least 3 bouts after the Dark-to-Light transition are adjusted, so this also does not violate Algorithm II [Lock/Flip].

For the “efficient” version, implementing Algorithm I+II+III+IV, the only addition is an array of probabilities that modifies the probability of LD0~LD1 flipping as a function of the preceding light interval length. This probability array is manually adjusted to fit the equivalent probability array for the VC assay (Figure 5F).

To be more specific, to implement Algorithms III and IV, (1) the angles were preliminarily determined only using Algorithms I+II; (2) then the left/right direction is either flipped or unchanged. In Algorithm III, step (2) assigns the direction so that the new turn is always (probability of 1) in the direction that favor the cumulative angle to approach (with opposing sign) the cumulative angle of the preceding dark period. In Algorithm IV, step (2) assigns the direction so that the new turn would be a “LD0~LD1 flip” with a probability that is equal to the corresponding probability from real fish (implemented in the code is essentially a 1-dimensional array of probabilities, as a function of time spent in light, taking the values as the red curve in Figure 5F).

## Results

### Temporal vs. spatial phototaxis

In order to test whether phototaxis consists of both spatial and temporal processing, we performed two simple assays: a spatial comparison assay and a temporal comparison assay. In the spatial comparison assay, a diffusive white screen surrounded by a black ring is placed beneath the arena (a transparent dish) and illuminated from below (Figure 1A). As expected, we find that a freely swimming fish stays within the (illuminated) center circle and avoids crossing into the dark area. We call this the “spatial comparison” assay because the fish can see the scattered light from the white area from all directions; as it is approaching the dark area, it can use this visual information to avoid crossing the border.

In the temporal comparison assay, named in the following the “Virtual Circle Assay” or “VC assay” (Figure 1B), we define a circular “virtual border” (that is invisible to the fish) in the center of a circular arena that is uniformly illuminated with white light through a diffusive screen. The fish starts inside the virtual circle, but as soon as it swims out of the circle, the light is switched off and the fish experiences complete darkness; when the fish returns, the whole field illumination is restored. Throughout the assay, the fish only perceives temporal changes of uniform white or uniform black visual inputs. We found that fish employ a variety of strategies to efficiently return to the virtual circle when plunged into darkness. The trajectory of the fish is therefore predominantly confined within the virtual border. This effect can also be observed when shapes other than a circle are used. Figure S1A shows examples of fish navigating in different virtual contexts with similar success.

The quantification of this behavior is greatly facilitated because larval zebrafish swim in discrete bouts (at a frequency of ~1 bout per second). A bout is characterized by a short burst of tail-oscillations, followed by an interbout period where the tail does not move (Budick and O'Malley, 2000). In our assay, the position and heading direction of the fish is tracked smoothly with a high-speed infrared-sensitive camera. Because the heading direction oscillates together with the tail, bouts can be robustly detected by thresholding the change in heading-directions (Figure 1C lower panel; the corresponding velocity is shown for comparison in the upper panel). The complex trajectory of the fish can thus be dissected into a chain of discrete bouts for quantitative analyses.

We are most interested in trajectory segments close to the virtual border (Figure 1D), since these comprise the immediate response to changes in illumination. The most prominent feature we observed is that after exiting the virtual circle and experiencing darkness, the fish quickly returns to the virtual circle. The average probability of returning within 3 bouts is 0.86 ± 0.02 (mean ± s.e.m, *n* = 32 fish, Figure 1E).

### Turning-angle distributions in light, dark, and at transitions

In order to explain this remarkably high success rate, we examined the fish's navigation strategies by tracking the angles between successive bouts (turning angles). While in light (within the virtual circle), fish mostly swim forward or make small angle turns, whereas after a light-to-dark transition, the average turning angle is increased significantly (Figure 2A). Larval zebrafish are known to respond to a dark flash with a large-angle turn (Burgess and Granato, 2007), but this single-step reflex alone does not ensure that they escape the dark area. If the fish is still in the dark after the first turn, subsequent turns are also important.

To investigate the fish's response over several turns, we summarized the turning angle distributions for each of several turns after light-to-dark or dark-to-light transitions for all fish (Figure 2B; 2 examples for single fish shown in Figure S2B). The results suggest that the determination of turning angle magnitudes is modified by the light-switching experience; the effect is strongest immediately after the switching and diminishes to baseline over at least 3 bouts.

The top histogram in yellow (left panel, first row) shows all turns performed in the light (*n* = 30464), and serves as an estimate of the baseline swimming activity. The top histogram in purple (right panel, first row) shows all turns performed in the dark (*n* = 10124). As noted previously, the first turn after the Light-to-Dark transition, labeled “LD1,” is typically a large angle turn. Subsequent turns in dark, “LD2” and “LD3,” are also large angle turns but their size decreases steadily (Figure 2C, purple line). Once the fish returns to the virtual circle, it must cease making large angle turns to avoid exiting it again. Indeed, the first turn after the Dark-to-Light transition, labeled “DL1,” is smaller than turns in dark, but still slightly larger than subsequent turns (Figure 2C, yellow line). Beyond 3 bouts in light or dark, turning angle magnitudes resemble those during baseline swimming activity (Figure 2B, bottom row).

We have thus described a set of relatively simple behavioral rules that modulates the turning angles directly based on the light transitions. We will henceforth refer to this set of rules as “Algorithm I [Angle]” to distinguish it from three further sets of rules which are discussed later. To test the functional relevance and contribution of these algorithms we built a simple computational model that allows us to test how much of the more complex features of the behavior these algorithms can explain.

Since larval zebrafish swim in discrete bouts, the model simulates the trajectory of a fish bout by bout. The dimensions of the simulated arena and virtual circle are designed to match the real VC assay. The displacement for a bout is approximately constant for real fish, and is held constant in the simulation. For each bout, the light state (on/off) is determined based on the updated position (in relationship to the virtual border in the simulation), and the turning angle of the next bout is determined by the algorithms.

This first version of the simulation implements Algorithm I [Angle], by drawing turning angles from different distributions depending on the light transitions. Figure 2D shows a representative sample trajectory each for the real fish and the simulation. To quantify and compare the degree that the fish trajectory is confined within the virtual border, we computed the average probability of the fish (real or simulated) returning to the light within 3 bouts (Figure 2E). A control simulation without specific algorithms is also performed, where all turning angles are drawn from a single generic turning-angle distribution (pooled from all turns from all fish). This control probability is only 0.05 ± 0.003 (mean ± s.e.m). With Algorithm I [Angle] implemented, the probability reaches 0.64 ± 0.006, which is still significantly smaller than the probability in real fish (0.86 ± 0.02, as shown in Figure 1E).

### History-dependent turning modulation around light transitions

We have shown that a fish responds to a sudden decrease in light intensity with a large angle turn. If the first large-angle turn does not lead the fish back to the virtual circle, it continues to make turns of large magnitudes. But are there rules for the left/right direction of these turns? For example, do fish continuously turn in one direction to return to the virtual circle, or do they turn randomly? To answer these questions, we analyzed the dependence of turn direction on the recent turning history. We found that fish are more likely to turn in the direction opposite to the previous turn immediately after a light transition, and tend to turn in the same direction otherwise.

For our analysis, we plotted a series of turning correlation matrices for different categories of turns to evaluate the relationship between a given turn and the turn immediately preceding it. The matrices are heat-map presentations of data summarized for all fish, normalized such that each fish is represented equally. For a set of two consecutive turns, the angles of the first turn and second turn are mapped onto the horizontal and the vertical axes, respectively.

In order to quantify this correlation, we also define a “lock-index,” which is positive for two consecutive turns in the same direction (“locked”) and negative for two turns in opposite directions (“flipped”). The value of the lock-index can range from −1 to 1, with 1 standing for maximally “locked” (identical turning magnitudes in the same direction) and −1 for maximally “flipped” (identical turning magnitudes but opposite direction). Figure 3A maps the values of the lock-index onto a correlation matrix. The average lock-indices for each fish are summarized in a histogram displayed above each of the correlation matrices.

Figure 3B shows the correlation of all pairs of consecutive turns in light (left panel) and in dark (right panel). In light, fish mostly perform small angle turns; in dark, turns are frequently of larger angle, but in both cases, pairs of consecutive turns are mostly correlated or “locked,” as also shown in the lock-index histograms on top of the matrices. We further examined the relationship between consecutive turns surrounding the light transitions (Figures 3E,F). Consider a sequence of events in which the fish leaves the border (into the dark) and then returns to the circle (back into light). (1), the turns spanning the Light-to-Dark transition (LD0 and LD1) are predominantly “flipped” (Figure 3E, left panel). (2), immediately after this Light-to-Dark transition, the first two bouts in dark (LD1 and LD2) are strongly “locked” large-angle turns (Figure 3E, right panel). (3), for the Dark-to-Light transition (Figure 3F), DL0 and DL1 are strongly “flipped,” and (4), DL1 and DL2 are “locked.” Effectively, it appears that the “lock” and “flip” turns could contribute to a fish staying close to the virtual border (as illustrated in Figure 3K). All together, we term these 6 groups of correlations between 2 consecutive turns “Algorithm II [Lock/Flip].”

### Playback experiments

To test whether these correlations depend on the shape of the virtual border, we conducted playback (Dark-Flash) experiments that essentially served as a yoked control for the VC assay. In the playback experiment, we deliver the temporal sequence of light transitions experienced by a fish in the VC assay to a different (naïve) animal. Specifically, we collected the statistics of light and dark durations that an animal experiences during the VC assay (Figure 3D), and draw from these two distributions to produce sequences of dark flashes to present to fish in the playback experiment (Figure 3H). In order to mimic the motion triggered nature of the VC assay, light changes are only delivered when the fish initiates a bout. Comparing the trajectories of the two experiments (Figures 3C,G), we observe that for playback, the locations at which large-angle turns occur is distributed randomly in space, and unsurprisingly, the overall trajectory is not confined. However, the “lock” and “flip” matrices generated from playback experiments are indistinguishable—in direction as well as magnitude (Figures 3I,J)—from the VC experiment described in Figures 3E,F (magnitude is also shown separately in Figure S3A). Together, the playback data show that these history-dependent turning modulations are robust and most likely innate features, which exist regardless of the existence of the virtual border.

If Algorithm II [Lock/Flip] is added to Algorithm I [Angle] in the simulation, fish show an improved localization within the virtual border, which generates a much closer match to the statistics of real fish (Figures 3L–N, compare to Figures 2D,E). Nevertheless, Algorithm II [Lock/Flip] alone, in the absence of Algorithm I [Angle], does not contribute significantly to the fish's performance (Figures S3B,C).

### Affinity to the virtual border can be simulated by turning-angle integration

**Figure 4:**
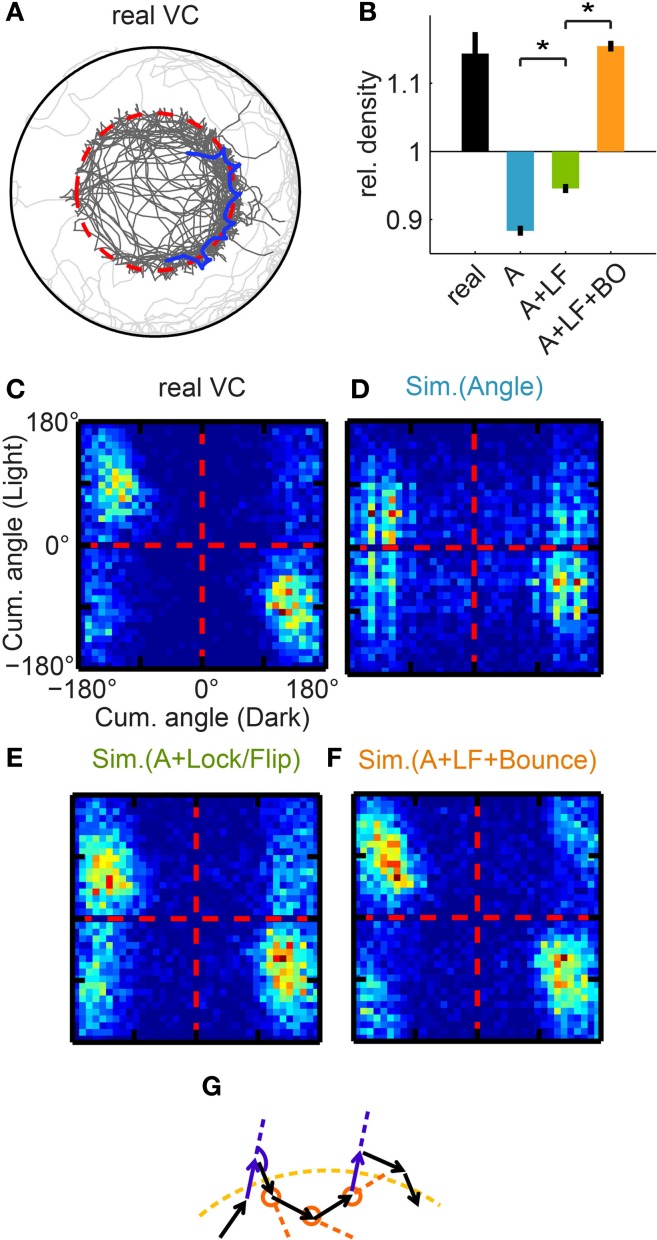
**Affinity to the virtual border. (A)** Trajectory of a real fish, example trajectory segment highlighted in blue. Note that the trajectory density is much higher close to the virtual border (dotted red line). **(B)** Quantification of the relative bout density close to the virtual border, and comparison between real fish and different simulations. Simulations are labeled by the algorithms implemented: “A” = Algorithm I [Angle], “LF” = Algorithm II [Lock/Flip], “BO” = Algorithm III [Bounce]. Reference level (=1) is the normalized baseline bout density if the trajectory were uniformly distributed within the Virtual Circle. (Quantification see Figure S4A) **(C–F)** Correlation between the cumulative angle turned during a light interval and during the preceding dark interval, again shown by matrices as in Figure 3B. **(C)** For real fish, strong clustering is shown close to the “flip” diagonal. **(D)** For the simulation with Algorithm I [Angle], there is no strong “lock”/“flip” bias. **(E)** In the simulation including Algorithm II [Lock/Flip], clustering in the “lock” quadrants is stronger than for real fish. **(F)** In the simulation including Algorithm III [Bounce], the simulated fish are constrained to match the “flip” pattern of real fish, and the resulting matrix confirms that the similarity to real fish is achieved. As shown in the last bar of **(B)**, the addition of this algorithm restores the high bout density of real fish in the simulation. **(G)** Illustration of Algorithm III [Bounce]. If the fish exits the virtual border at approximately the same angle (relative to the border) each time, the fish may frequently cross the virtual border. That would require the heading direction of the two purple bouts to be approximately parallel, and the angle of the turn in dark (marked with the purple arc) should have equal magnitude but opposite direction as the sum of the 3 following turns in light (marked with orange circles). ^*^p < 0.001.

The pooled trajectories of the VC assay revealed a “border hugging” feature (Figure 4A) that was not captured by the simulation. The fish seems to repeatedly exit and enter the virtual circle as it navigates along the virtual border, almost as if it were “bouncing” off the border (highlighted blue trajectory segment). To quantify and compare this enhanced activity near the border, we first calculate the relative trajectory density near the virtual border (Figure 4B, first 3 bars). For real fish, this relative density at the border (1.14 ± 0.03, mean ± s.e.m, *n* = 32) is significantly larger than expected from a uniform distribution (see Figure S4A). The density for the simulation with Algorithm I+II (0.95 ± 0.007, *n* = 100) is slightly higher than for the simulation with Algorithm I alone (0.88 ± 0.007, *n* = 100), but is still significantly lower than for real fish. We therefore searched for an algorithm that would recapture this high density at the border. We know that Algorithm I and II only use information of the previous one bout in the swimming history. Thus, we tested whether the inclusion of more than one bout in history leads to an improvement of the simulation.

As such, Algorithm III [Bounce] was inspired by analyzing the cumulative turning angle over all successive bouts between light transitions. For real fish, the cumulative angle turned over a given light interval is “flipped” in relation to the cumulative angle turned over the preceding dark interval (Figure 4C). This pattern is not well captured in the previous versions of the simulation (Figures 4D,E). In Algorithm III, each turn in light is therefore biased such that their cumulative angle approaches (with opposing sign) the cumulative angle of the preceding dark period. The implementation of Algorithm III resulted in a cumulative correlation matrix that closely resembles that of real fish (compare Figure 4F and 4C). Importantly, in the presence of previous algorithms (Figure S4C), this simulation fully recaptures the “border-hugging” feature described in Figure 4B (last bar: 1.15 ± 0.008, mean ± s.e.m, *n* = 100; trajectory shown Figure S4B).

Figure 4G gives an intuition for how the flipping of the cumulative angles leads to a trajectory that frequently crosses the virtual border: the underlying pattern is that the trajectory curve on one side of the virtual border is loosely mirrored on the opposite side. Also note that in the correlation matrix, the data points strongly cluster between the “flip” (anti-correlation) diagonal and the horizontal axis, demonstrating that as expected for a circle (instead of a straight line), the cumulative angle turned in light is usually slightly smaller in magnitude than in dark.

### Choice of efficient turning direction suggests sophisticated navigation ability

**Figure 5:**
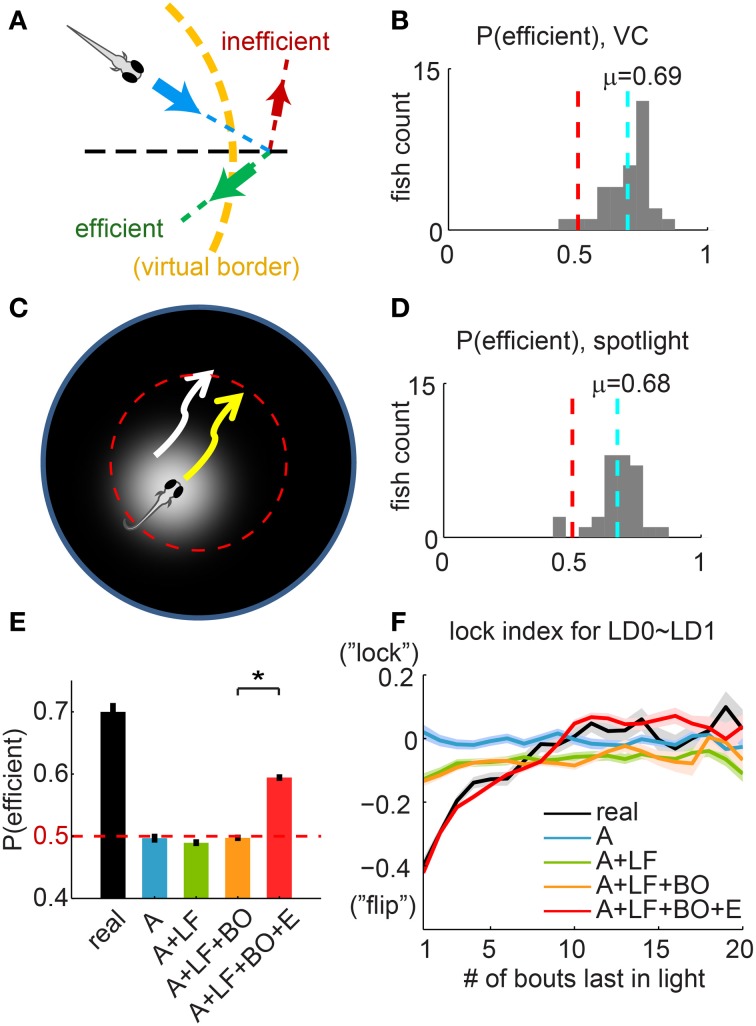
**Choice of efficient turning direction suggests sophisticated navigation ability. (A)** Illustration of “efficient” vs. “inefficient” turns. For a fish approaching the virtual border (dashed yellow line) along the direction indicated by the blue arrow, in order to return to the light, a turn in one direction (green arrow) is more “efficient” than in the other direction (red arrow). Dashed black line: radial direction. **(B)** Histogram of the per-fish “efficiency,” summarized for all fish in the VC assay. The 50/50 probability (pure chance) is indicated with a dashed red line. The dashed cyan line marks the mean of the distribution (0.69 ± 0.016, mean ± s.e.m, *n* = 32). **(C)** “Spotlighted” Virtual Circle experiment, to control for potential asymmetries in the visual field that can be used as visual cues. The projected spotlight is centered at the fish at all times, except when the fish exits the virtual border (dashed red line) and the light is turned off. **(D)** Histogram of the per-fish “efficiency”, for 30 fish from the “Spotlighted” Virtual Circle experiment. The mean of this population (0.68 ± 0.016, mean ± s.e.m, *n* = 30) is unchanged compared to **(B)**. **(E)** The average “efficiency” compared between real fish and different simulations. Simulations are labeled by all the algorithms implemented: “A” = Algorithm I [Angle], “LF” = Algorithm II [Lock/Flip], “BO” = Algorithm III [Bounce], “E” = Algorithm IV [Efficiency]. Dashed red line indicates the value of pure chance. None of the first 3 simulations (blue, green, and orange) produce an “efficiency” that is statistically different from chance. Only the simulation applying Algorithm IV, as described in **(F)**, enhances the “efficiency” significantly to 0.59 ± 0.005 (mean ± s.e.m). **(F)** The lock-index for the last turn in light (LD0) and first turn in dark (LD1), plotted as a function of the length of the immediately preceding interval in light. Note that for the previous simulations, the lock-index does not change significantly with the length of the preceding light interval. For the simulation with Algorithm IV [Efficiency], the turning direction of the first turn in dark (LD1) is constrained so that this lock-index curve (in red) mimics the curve from real fish (in black). ^*^p < 0.001.

If we only consider the first turn after the Light-to-Dark transition (LD1), in most cases, turning left or right is not equally efficient for returning to the virtual circle (Figure 5A). For a fish approaching the virtual border at an angle indicated by the blue arrow, we count a turn in the direction of the green arrow as an “efficient” turn, and one in the direction of the red arrow as an “inefficient” one. To quantify this “efficiency” for a given fish, we calculate the probability that an LD1 turn is in the “efficient” direction. Given that the virtual border is completely invisible to the fish, if the fish were to make turns in a random direction, the average “efficiency” would be 0.5.

To our surprise, we found that for the population of fish tested, fish turn into the “efficient” direction 70% of the time (Figure 5B, “efficiency” = 0.69 ± 0.02, mean ± s.e.m, *n* = 32). In order to control for residual spatial visual cues as a possible explanation for this phenomenon, we designed a “Spotlighted Virtual Circle” experiment (Figure 5C). A white circle (the “spotlight”) is projected onto the otherwise black screen and is always centered at the fish while the fish is swimming within the virtual border. Here again, the projector is turned off and the arena is left in complete darkness when the fish exits the border. We found that the probability of “efficient” turns is unaffected by these more stringent conditions (Figure 5D, 0.68 ± 0.02, mean ± s.e.m). Therefore, we can conclude that the fish is not informed by spatial visual cues in the VC assay to guide its behavioral choices.

The three algorithms described thus far do not reproduce this “efficiency” (Figure 5E, first 4 bars; mean ± s.e.m for Angle: 0.50 ± 0.007; Angle + Lock/Flip: 0.49 ± 0.005; Angle + Lock/Flip + Bounce: 0.50 ± 0.005). Therefore we added another algorithm, Algorithm IV [Efficiency], to fully explain the observed behavior. In this algorithm, the strong tendency of fish to “flip” between the last turn in light (LD0) and the first turn in dark (LD1) is relaxed depending on the time spent in light (number of bouts executed) before this transition into dark. Indeed, we find that in real fish a “lock” or a “flip” is equally likely if the last interval in light lasted for many (≳10) bouts, a feature that is not captured by previous algorithms (Figure 5F). Intuitively, if the fish swam for a long time within the circle, it will approach the border at more random angles and a “flip” strategy is not likely to be more “efficient” than chance.

Algorithm IV did not offset the overall LD0~LD1 lock-index (as shown in the correlation matrix in Figure S5A), but its addition to the simulation resulted in a significant increase of the “efficiency” (0.59 ± 0.005, mean ± s.e.m, Figure 5E, last bar), which accounts for half of the difference between real fish and chance level. Additionally, we show in Figure S5B that Algorithm III contributes indirectly to the “efficiency” (the “efficiency” is compromised to 0.56 ± 0.006 in its absence). We also examined the lock-index trend for the 2 bouts around the entry to the light, and there was no significant dependence on the length of the preceding dark interval (Figure S5C).

One additional explanation for this surprising ability of the fish to turn into the correct direction might be that the fish—by dead reckoning—accumulates information about the actual location of the virtual circle throughout the assay and then uses this information to gradually improve efficiency. In order to test this hypothesis, we compared the fish's performance at the very beginning of the VC assay with its overall performance. The efficiency calculated from the first 2 min of each session (0.72 ± 0.02, mean ± s.e.m; Figures S5D–F) is already indistinguishable from the overall value. This suggests that while the fish must integrate some simple information about the recent swimming history to make efficient choices, it is not required for the animal to form a spatial representation of the virtual circle.

## Discussion

To the best of our knowledge, this is the first study to explore the temporal aspects of phototaxis in the larval zebrafish. Here we extract a series of simple temporal algorithms that explain most of the animal's behavioral statistics. These algorithms range from hard-wired turning modulations to temporal integration strategies that suggest advanced navigation abilities. We were able to extract these algorithms due to two key features of the Virtual Circle assay. First, the visual input is a series of binary events (Light/Dark) in time; in other words, the visual information is encoded purely temporally, as it is spatially uniform at all times. Second, larval zebrafish swimming can be described in discrete bouts, which enables us to describe the algorithms in discrete behavioral units.

The visual input does not provide direct spatial information, yet the fish can avoid dark areas in space; therefore the fish must rely on temporal integrations, i.e., some form of memory, to implement the 4 behavioral algorithms discussed in Figures 2–5, respectively. Here we summarize the 4 memory requirements for the VC assay in Table 1. (1) Time since light transition. The first memory requirement is the retention of light-switching events. For the turning-angle distributions (Algorithm I), the effect of the light switches lasts over several bouts (bout frequency is ~1 per second). For Algorithm IV, which enhances the efficiency, the gradual decay of the “flip” tendency for the first turn in Dark suggests retention of the light-switching event over up to 10 bouts (~10 sec). (2) Direction of last turn. Larval zebrafish are able to retain the turning direction of the previous turn to inform the choice of direction of the present turn. This is supported by Algorithm II, the lock/flip correlation of the turning angles of two consecutive turns. Algorithm IV [Efficiency] also depends on this memory requirement, since it is based on the “flip” around the light-off transition. (3) Cumulative angle. This form of memory is required by Algorithm III [Bounce], which ensures that the cumulative (total) angle made in a light interval is similar in amplitude (but in the opposite direction) as the cumulative angle in the preceding dark interval. (4) Higher order (spatial processing). In the final figure, the unexpected “efficiency” of the fish in returning to the virtual circle is only partially explained by simulations using relatively simple algorithms. The unaccounted part of the “efficiency” may invite thoughts on path integration (Müller and Wehner, 1988) or spatial memory, but a more likely scenario would be one that involves additional simple algorithms of the sort described in this study.

**Table 1:**
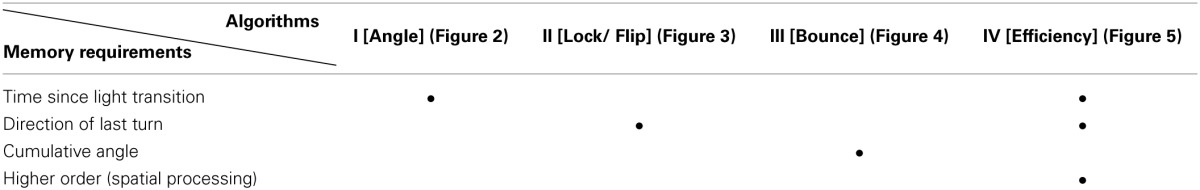
**Summary of behavioral Algorithms and the corresponding memory requirements**.

Under natural conditions, fish will usually not experience sharp step functions in light intensity. Rather, transitions are likely to be of a more gradual nature. To test whether this would affect the general features of the behaviors described here, we implemented temporal light gradients that may better represent natural light stimuli. In this gradient version of the VC assay, the uniform illumination dims gradually as the fish approaches the virtual border. We find that here also fish exhibit general turning behavior that results in high occupancy within the virtual border (Figure S1B). This serves as a proof of principle that larval zebrafish can use broadly effective temporal navigation strategies for temporal gradients of varied steepness. In addition, we show that a real ring-shaped shadow (as compared to a virtual one), also confines the fish to the center area in a very similar way (Figure S1C).

For isolated dark flashes in larval zebrafish, Burgess et al. reported turning angles of 150° ± 30° (mean ± *SD*) (Burgess and Granato, 2007), while for our VC assay, the LD1 turning angles are 113° ± 42°, significantly smaller in magnitude. However, in the VC assay, dark flashes occur more frequently (averaging once every ~8 s) than in Burgess et al. which might result in habituation effects that could explain this discrepancy (Figure S2A). Indeed, when we analyze the initial response strength in the VC assay we find that these turns show no difference to those reported by Burgess et al. (144° ± 31° for the turn after the first dark flash for each session, mean ± *SD*).

Upon closer inspection of Figure 2B, we also observe a trimodal distribution of turns in light: slightly left, straight forward, and slightly right (Figure 2B left column). Given the lack of directional stimuli in our assay, this swimming pattern may support a locomotion control model that distinguishes a forwards swimming mode from a turning mode (Huang et al., 2013).

For most of this study, we presented pooled data from all animals, and one may question whether individual left/right turning biases may contribute to features like the “border hugging.” We therefore manipulated the left/right bias of all turns in a simulation (that includes Algorithms I, II but not the “Bounce” Algorithm III). We found that while a turning bias of medium strength does not affect the bout density at the border, a strong bias actually decreases this density (Figure S4D), which argues against individual left/right biases being an underlying cause.

Fernandes et al. (2012) described a related light-seeking behavior in blind zebrafish larvae via deep brain photoreceptors as “an undirected hyperactivity in darkness, which results in the aggregation of organisms into a lit area,” and termed it “dark photokinesis.” This finding begs the question of whether the temporal phototaxis in our study could result from such simple diffusional trapping. The main argument against that is that undirected hyperactivity in darkness is a highly inefficient method for light-seeking, an observation confirmed by the analysis of Fernandes et al. In contrast, the phototactic behavior observed in our experiments shows remarkable efficiency that requires more complex rules.

Most of the behavioral features that emerged out of the VC assay serve to better avoid darkness, but the enhanced activity close to the virtual border (Figure 4) seems to be an exception and calls for a novel explanation of its adaptive advantage. We speculate that this frequent crossing of the border is of exploratory nature, and can help an animal to escape from the confinements of aversive stimuli (shadows). Such exploratory tendencies are well recognized in other animals; in rodents for instance, an increase of exploratory behavior is used as an indicator of decreased anxiety (Crawley, 1985).

The majority of the temporal algorithms discovered in larval zebrafish are likely innate, as opposed to learned. The playback experiments (Figures 3G–J, S3A), as well as the lack of performance improvement over time within one session (Figure S5F) strongly support this notion. Furthermore, we used very young animals (5–7 days old), and although they already demonstrate a rich repertoire of behaviors, associative learning for 5-day-old larvae is very difficult at best. This suggests that these temporal strategies are not shaped by experience and that fish employ these very same strategies in navigating their habitat around natural shadows.

Finally, the larval zebrafish is well suited to further dissect these kinds of behaviors at the neuronal level (Portugues and Engert, 2009; Friedrich et al., 2010; McLean and Fetcho, 2011) since it lends itself readily to whole-brain functional imaging at single-cell resolution (Ahrens et al., 2012, 2013a) and optical monitoring and manipulation of neural activity in a behaving animal (Douglass et al., 2008; Orger et al., 2008; Arrenberg et al., 2009; Wyart et al., 2009; Chow et al., 2010; Schoonheim et al., 2010; Warp et al., 2011; Akerboom et al., 2012; Ahrens et al., 2013b).

## Author contributions

Xiuye Chen designed and performed the experiments, analyzed the data and wrote the simulations. Xiuye Chen and Florian Engert conceived the project, discussed the data, and wrote the manuscript.

## Conflict of interest statement

The authors declare that the research was conducted in the absence of any commercial or financial relationships that could be construed as a potential conflict of interest.

## Supplementary material

Figure S1 **Variants of the Virtual Circle assay.** Related to Figure 1. **(A)** Shape variants of the VC assay. Left: a square-shaped virtual border. Example trace from a single fish shows that the trajectory is tightly restricted to within the border. Right: a more irregularly shaped virtual border. Traces from two fish are overlaid (note that each fish only reached the edge of the arena once). Insets: shape of virtual borders, with white area indicating location for (uniform) light-on, and dark areas for light-off. **(B)** Variant of VC assay with a temporal light gradient at the border (as opposed to a sharp transition). The ring-shaped area between the two dotted white circles is virtually defined as the transition zone: the illumination of the arena is spatially uniform at all times but changes in intensity while the fish is within the transition zone. When the fish is within the inner dotted circle, illumination is at the brightest; as the fish approaches the outer circle, the illumination dims, and gradually transitions into complete darkness when the fish reaches or is beyond the outer circle. Note that fish usually turns around before reaching complete darkness. **(C)** Larval zebrafish prefer light over real shadows (mostly temporal darkening). Upper left panel: the light source comes from below the arena, and a ring-shaped piece of material that blocks visible light (but transmits infrared light) is positioned under the arena, i.e., a strong shadow is created for the ring-shaped area. Lower left panel: trajectory of a fish over a session of 6 min. The fish mainly stays within the center circle of the dish where they can see the light source; when it crosses the border into the shadow area it quickly returns back to the circle. Insets show 3 trajectory segments (rotated into same orientation), direction indicated by the red, green and blue arrowhead, yellow dots mark the points where fish enters the shadow (crossing the border that is marked by dashed red line). Note similarity to the VC assay.

Figure S2 **Related to Figure 2. (A)** Magnitude of turning angle of first turn after Dark shows slow decay over time. There is a decay of the response strength to Dark Flashes due to habituation. Most sessions were conducted for 15 min, but even toward the end of the longer sessions (30 min), on average the fish still responds with large angle turns to the onset of Dark Flashes. **(B)** Sample turning angles distributions for individual fish, compare to Figure 2B. Two examples are shown in 2 rows, respectively.

Figure S3 **Related to Figure 3. (A)** Turning angle distribution from playback experiments; compare to Figure 2B. **(B)** Comparison of probability of returning to the virtual circle within 3 bouts, augmenting Figure 3L. We show a new simulation, “LF” (yellow bar), that implements the generic turning-angle distribution as in the control simulation, plus Algorithm II [Lock/Flip]. The resulting probability of returning within 3 bouts (0.07 ± 0.004, mean ± s.e.m) is barely higher than in the control simulation. This shows that Algorithm I [Angle] is necessary for later simulations incorporating Algorithm II. **(C)** Compare to Figure 3N, color-coded as in **(B)**.

Figure S4 **Related to Figure 4. (A)** Radial bout density for VC assay and simulations, supplementing Figure 4B. Horizontal axis: distance from center (radius), “R” notes the radius of the virtual border, “edge” the radius of the arena. Red line indicates the reference bout density as a function of radius, assuming uniform density of bouts within the virtual border and zero outside. The bout density ρ is defined for the cyan shaded range as the ratio of the simulated value (area in gray) to the reference value (area under red curve). To avoid systemic biases because of poor confinement to the virtual border (as in the simulation only with Algorithm I [Angle]), the bouts from trajectory segments where the fish did not return to the virtual border within 3 bouts are not included for this quantification. **(B)** Example trajectory of the simulation implementing Algorithm III [Bounce]. **(C)** Quantification of the relative bout density close to the virtual border, augmenting Figure 4B. Simulations are labeled by the Algorithms implemented. The purple bar shows that Algorithm II [Lock/Flip] still contributes significantly in the presence of Algorithm III [Bounce]. **(D)** Simulated relative density close to the virtual border, with the ratio of left vs. right turns manipulated (this simulation includes Algorithms I and II, but not III). Zero on the horizontal axis indicates all turns are right turns, and 1 on the axis indicates an equal mix of left/right turns. Positive biases for left turns (values >1 on the horizontal axis) are symmetrical but not shown.

Figure S5 **Related to Figure 5. (A)** Correlation matrix of LD0~LD1 for the simulation with Algorithm IV [Efficiency] is very similar to the matrix from real fish (Figure 3E first panel). **(B)** The average “efficiency” compared between real fish and different simulations, augmenting Figure 5E. Since Algorithm IV [Efficiency] depends on the “flip” of LD0~LD1, Algorithm II [Lock/Flip] is not permutated separately from Algorithm IV. The brown bar suggests that even though Algorithm III [Bounce] is not sufficient to enhance the “efficiency” alone, it still contributes significantly in the presence of Algorithm IV. **(C)** Similar to Figure 5F but for DL0 (last turn in dark) vs. DL1 (first turn in light). No significant change is seen in the real fish (black curve), and matching the black curve better (red curve) does not have significant effects on the simulation. **(D–F)** Data analyzed for only the first 2 min from all VC sessions. [For first 2 min of each session, P(within 3 bouts) = 0.96 ± 0.01, mean ± s.e.m, not shown in figure]. **(D)** Turning angle distributions in light (left) and dark (right); compare to Figure 2B. **(E)** Correlation matrix of cumulative angles; compare to Figure 4C. **(F)** Histogram of the per-fish “efficiency” (0.72 ± 0.02, mean ± s.e.m), compare to Figure 5B.
