# Supplementary figures and images for: Navigational strategies underlying phototaxis in larval zebrafish
Source: Front Syst Neurosci. 2014 Mar 25;8:39. doi: 10.3389/fnsys.2014.00039 (PMC3971168; doi:10.3389/fnsys.2014.00039)

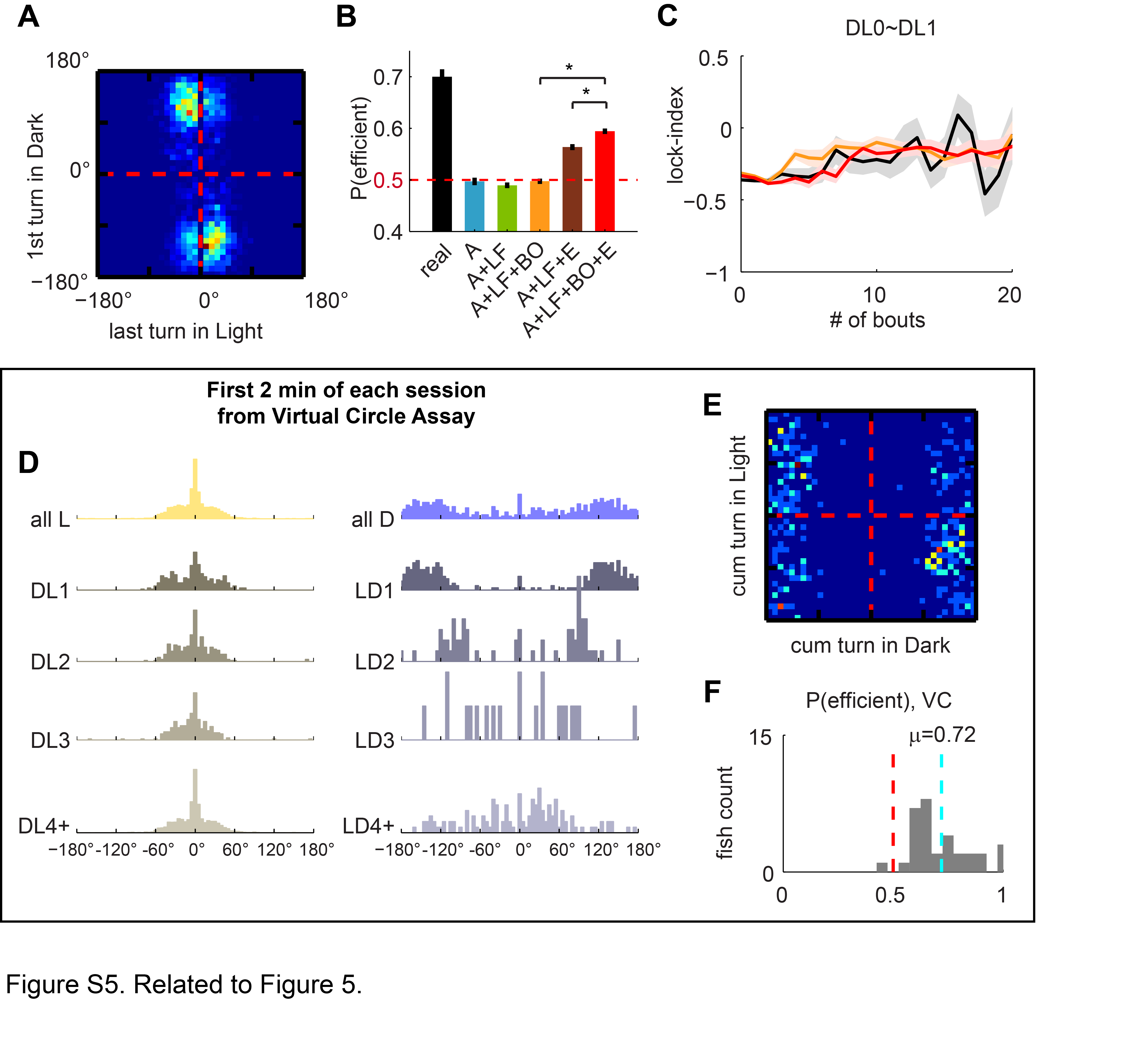

Supplement: Figure S1 — Variants of the Virtual Circle assay. Related to Figure 1. (A) Shape variants of the VC assay. Left: a square-shaped virtual border. Example trace from a single fish shows that the trajectory is tightly restricted to within the border. Right: a more irregularly shaped virtual border. Traces from two fish are overlaid (note that each fish only reached the edge of the arena once). Insets: shape of virtual borders, with white area indicating location for (uniform) light-on, and dark areas for light-off. (B) Variant of VC assay with a temporal light gradient at the border (as opposed to a sharp transition). The ring-shaped area between the two dotted white circles is virtually defined as the transition zone: the illumination of the arena is spatially uniform at all times but changes in intensity while the fish is within the transition zone. When the fish is within the inner dotted circle, illumination is at the brightest; as the fish approaches the outer circle, the illumination dims, and gradually transitions into complete darkness when the fish reaches or is beyond the outer circle. Note that fish usually turns around before reaching complete darkness. (C) Larval zebrafish prefer light over real shadows (mostly temporal darkening). Upper left panel: the light source comes from below the arena, and a ring-shaped piece of material that blocks visible light (but transmits infrared light) is positioned under the arena, i.e., a strong shadow is created for the ring-shaped area. Lower left panel: trajectory of a fish over a session of 6 min. The fish mainly stays within the center circle of the dish where they can see the light source; when it crosses the border into the shadow area it quickly returns back to the circle. Insets show 3 trajectory segments (rotated into same orientation), direction indicated by the red, green and blue arrowhead, yellow dots mark the points where fish enters the shadow (crossing the border that is marked by dashed red line). Note similarity to [file Presentation1.ZIP › 81220_Engert_Supplementary Figure_5.TIF]

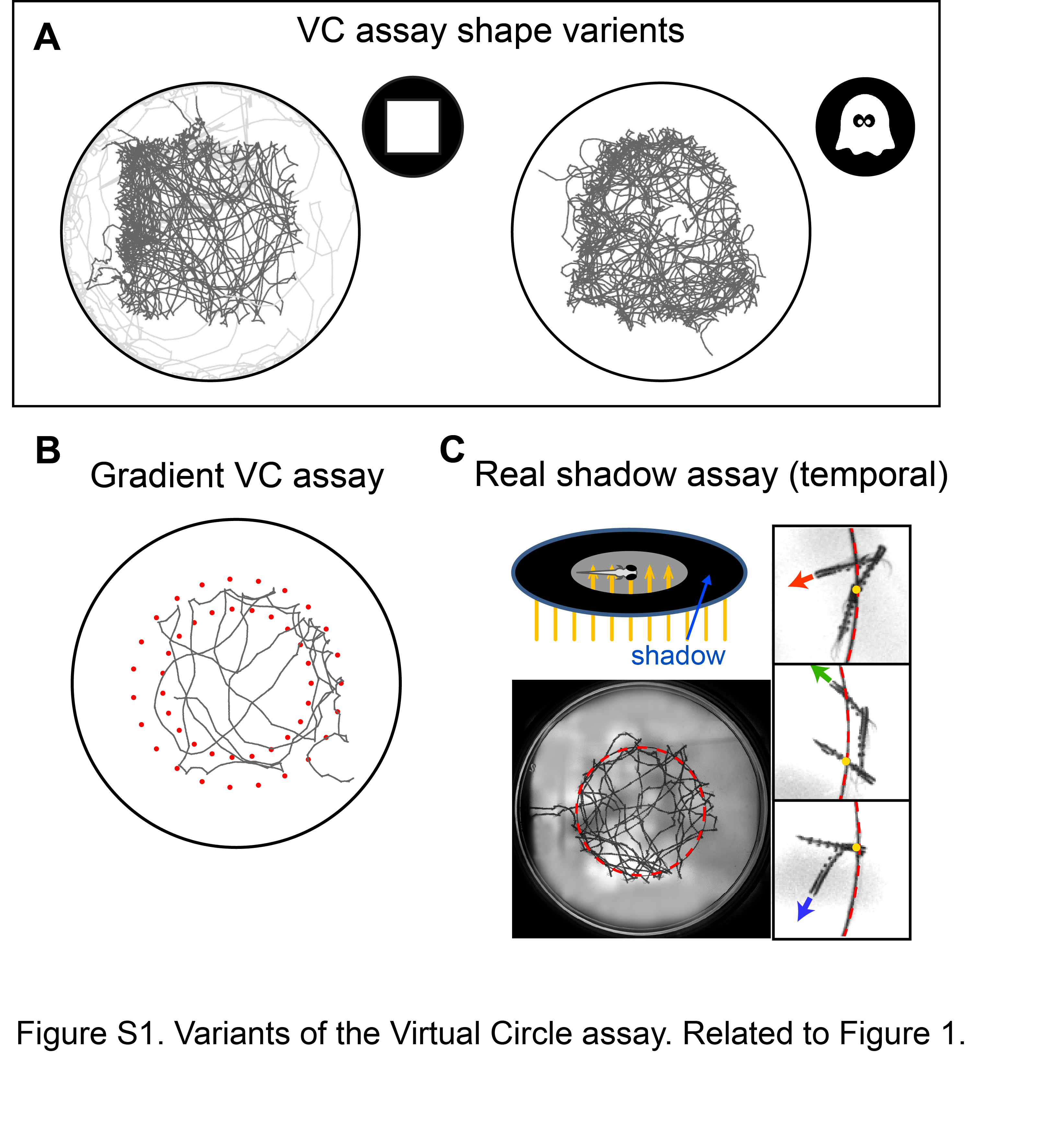

Supplement: Figure S1 — Variants of the Virtual Circle assay. Related to Figure 1. (A) Shape variants of the VC assay. Left: a square-shaped virtual border. Example trace from a single fish shows that the trajectory is tightly restricted to within the border. Right: a more irregularly shaped virtual border. Traces from two fish are overlaid (note that each fish only reached the edge of the arena once). Insets: shape of virtual borders, with white area indicating location for (uniform) light-on, and dark areas for light-off. (B) Variant of VC assay with a temporal light gradient at the border (as opposed to a sharp transition). The ring-shaped area between the two dotted white circles is virtually defined as the transition zone: the illumination of the arena is spatially uniform at all times but changes in intensity while the fish is within the transition zone. When the fish is within the inner dotted circle, illumination is at the brightest; as the fish approaches the outer circle, the illumination dims, and gradually transitions into complete darkness when the fish reaches or is beyond the outer circle. Note that fish usually turns around before reaching complete darkness. (C) Larval zebrafish prefer light over real shadows (mostly temporal darkening). Upper left panel: the light source comes from below the arena, and a ring-shaped piece of material that blocks visible light (but transmits infrared light) is positioned under the arena, i.e., a strong shadow is created for the ring-shaped area. Lower left panel: trajectory of a fish over a session of 6 min. The fish mainly stays within the center circle of the dish where they can see the light source; when it crosses the border into the shadow area it quickly returns back to the circle. Insets show 3 trajectory segments (rotated into same orientation), direction indicated by the red, green and blue arrowhead, yellow dots mark the points where fish enters the shadow (crossing the border that is marked by dashed red line). Note similarity to [file Presentation1.ZIP › 81220_Engert_Supplementary Figure_1.TIF]

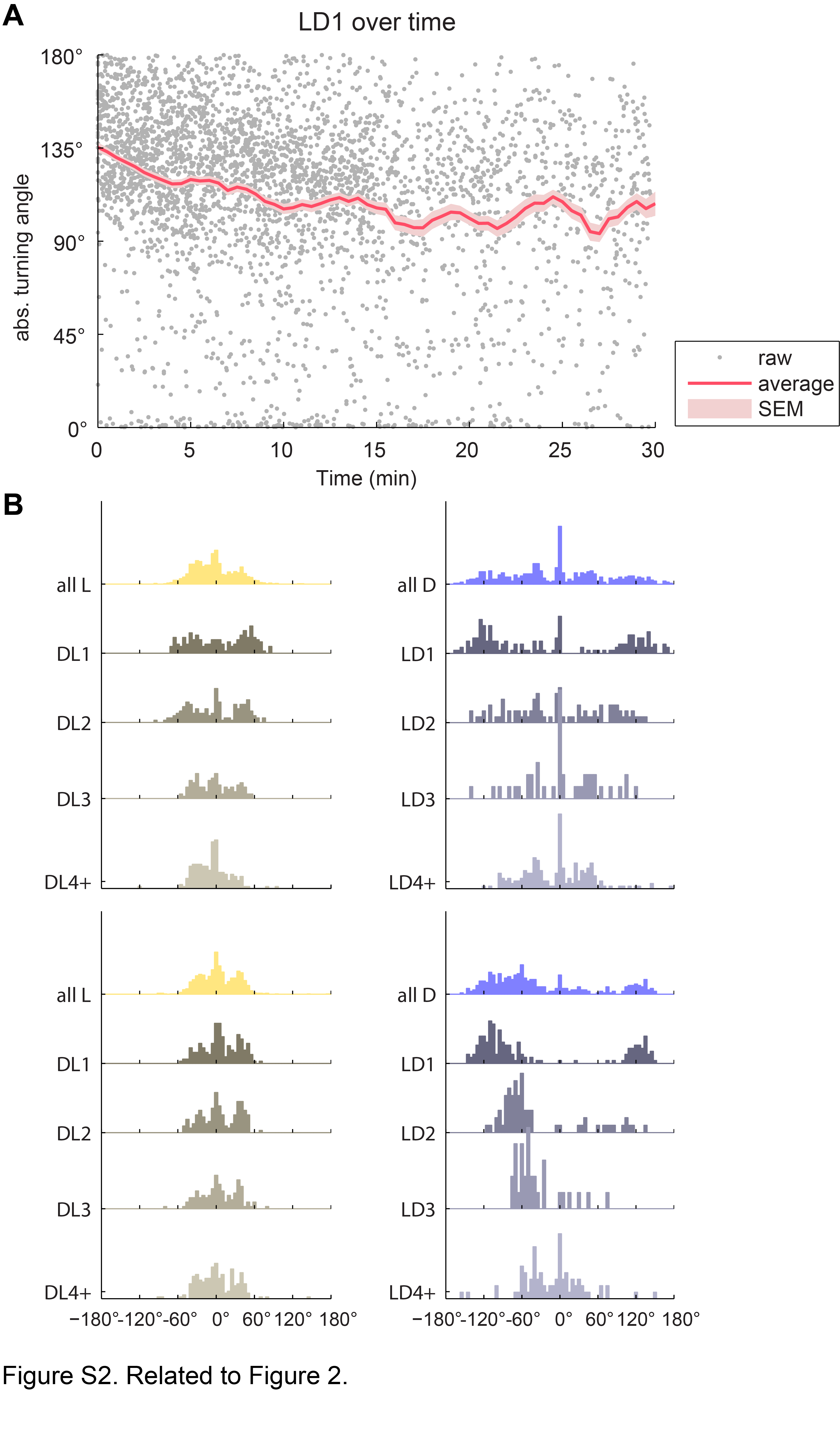

Supplement: Figure S1 — Variants of the Virtual Circle assay. Related to Figure 1. (A) Shape variants of the VC assay. Left: a square-shaped virtual border. Example trace from a single fish shows that the trajectory is tightly restricted to within the border. Right: a more irregularly shaped virtual border. Traces from two fish are overlaid (note that each fish only reached the edge of the arena once). Insets: shape of virtual borders, with white area indicating location for (uniform) light-on, and dark areas for light-off. (B) Variant of VC assay with a temporal light gradient at the border (as opposed to a sharp transition). The ring-shaped area between the two dotted white circles is virtually defined as the transition zone: the illumination of the arena is spatially uniform at all times but changes in intensity while the fish is within the transition zone. When the fish is within the inner dotted circle, illumination is at the brightest; as the fish approaches the outer circle, the illumination dims, and gradually transitions into complete darkness when the fish reaches or is beyond the outer circle. Note that fish usually turns around before reaching complete darkness. (C) Larval zebrafish prefer light over real shadows (mostly temporal darkening). Upper left panel: the light source comes from below the arena, and a ring-shaped piece of material that blocks visible light (but transmits infrared light) is positioned under the arena, i.e., a strong shadow is created for the ring-shaped area. Lower left panel: trajectory of a fish over a session of 6 min. The fish mainly stays within the center circle of the dish where they can see the light source; when it crosses the border into the shadow area it quickly returns back to the circle. Insets show 3 trajectory segments (rotated into same orientation), direction indicated by the red, green and blue arrowhead, yellow dots mark the points where fish enters the shadow (crossing the border that is marked by dashed red line). Note similarity to [file Presentation1.ZIP › 81220_Engert_Supplementary Figure_2.TIF]

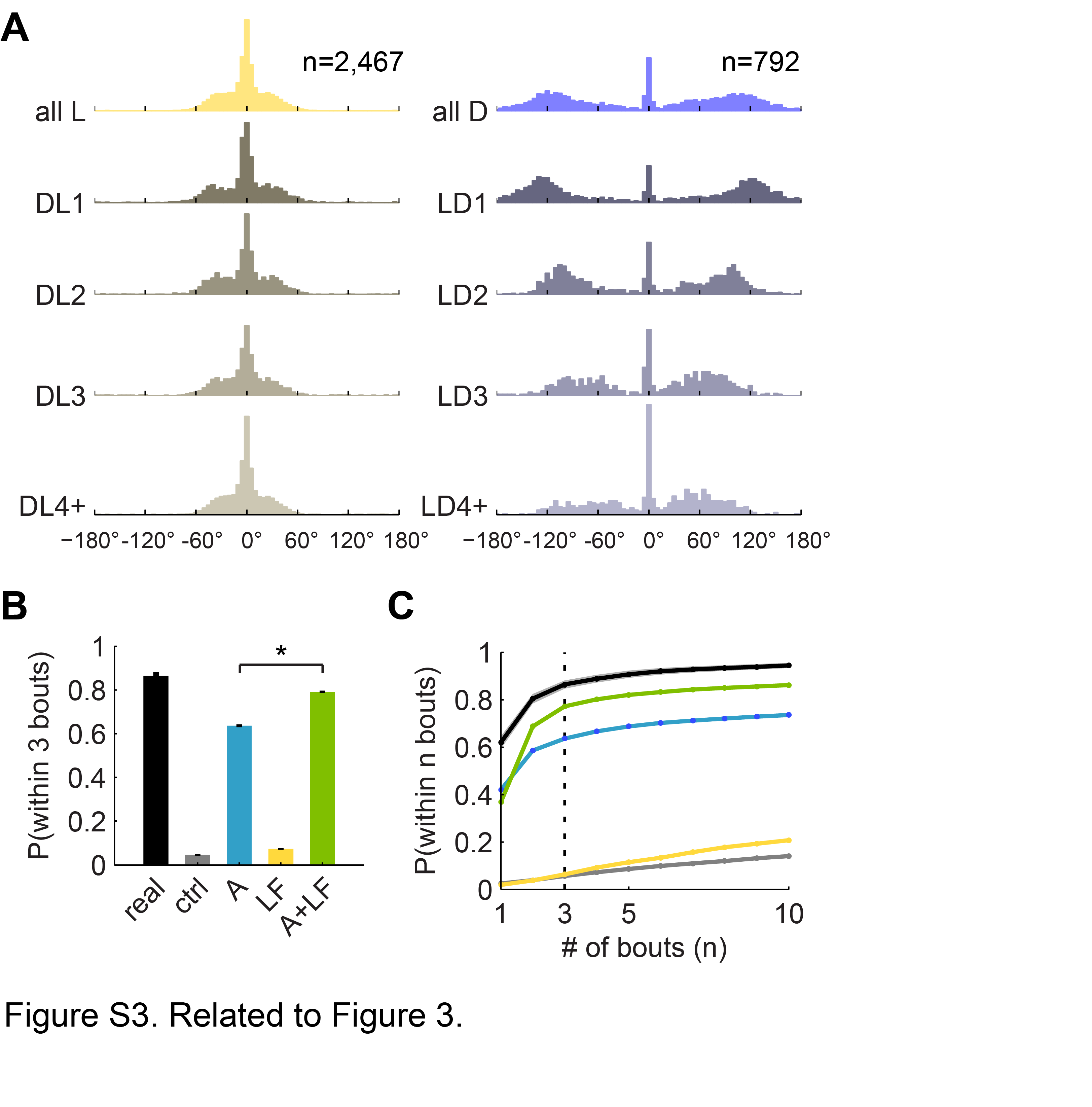

Supplement: Figure S1 — Variants of the Virtual Circle assay. Related to Figure 1. (A) Shape variants of the VC assay. Left: a square-shaped virtual border. Example trace from a single fish shows that the trajectory is tightly restricted to within the border. Right: a more irregularly shaped virtual border. Traces from two fish are overlaid (note that each fish only reached the edge of the arena once). Insets: shape of virtual borders, with white area indicating location for (uniform) light-on, and dark areas for light-off. (B) Variant of VC assay with a temporal light gradient at the border (as opposed to a sharp transition). The ring-shaped area between the two dotted white circles is virtually defined as the transition zone: the illumination of the arena is spatially uniform at all times but changes in intensity while the fish is within the transition zone. When the fish is within the inner dotted circle, illumination is at the brightest; as the fish approaches the outer circle, the illumination dims, and gradually transitions into complete darkness when the fish reaches or is beyond the outer circle. Note that fish usually turns around before reaching complete darkness. (C) Larval zebrafish prefer light over real shadows (mostly temporal darkening). Upper left panel: the light source comes from below the arena, and a ring-shaped piece of material that blocks visible light (but transmits infrared light) is positioned under the arena, i.e., a strong shadow is created for the ring-shaped area. Lower left panel: trajectory of a fish over a session of 6 min. The fish mainly stays within the center circle of the dish where they can see the light source; when it crosses the border into the shadow area it quickly returns back to the circle. Insets show 3 trajectory segments (rotated into same orientation), direction indicated by the red, green and blue arrowhead, yellow dots mark the points where fish enters the shadow (crossing the border that is marked by dashed red line). Note similarity to [file Presentation1.ZIP › 81220_Engert_Supplementary Figure_3.TIF]

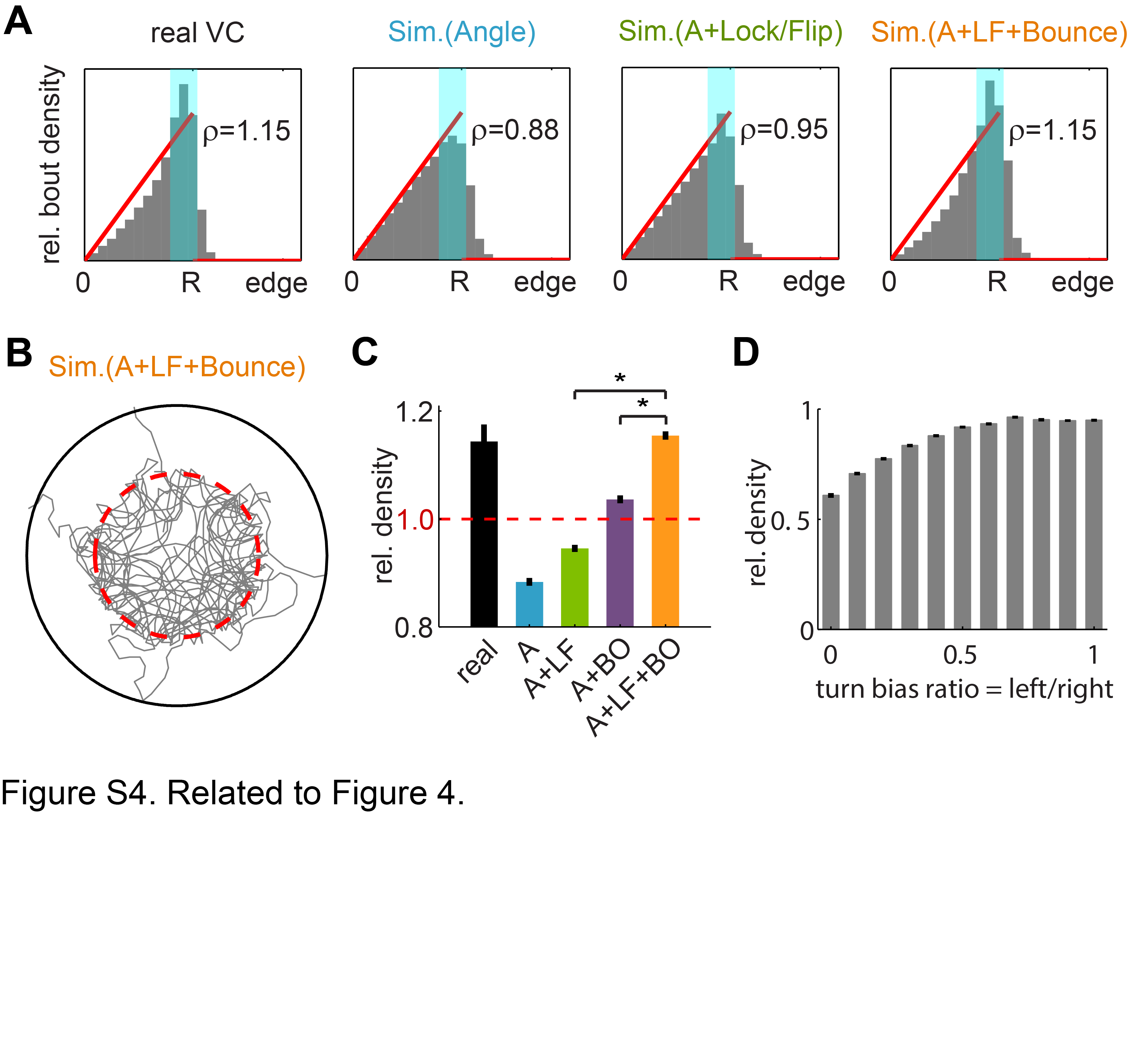

Supplement: Figure S1 — Variants of the Virtual Circle assay. Related to Figure 1. (A) Shape variants of the VC assay. Left: a square-shaped virtual border. Example trace from a single fish shows that the trajectory is tightly restricted to within the border. Right: a more irregularly shaped virtual border. Traces from two fish are overlaid (note that each fish only reached the edge of the arena once). Insets: shape of virtual borders, with white area indicating location for (uniform) light-on, and dark areas for light-off. (B) Variant of VC assay with a temporal light gradient at the border (as opposed to a sharp transition). The ring-shaped area between the two dotted white circles is virtually defined as the transition zone: the illumination of the arena is spatially uniform at all times but changes in intensity while the fish is within the transition zone. When the fish is within the inner dotted circle, illumination is at the brightest; as the fish approaches the outer circle, the illumination dims, and gradually transitions into complete darkness when the fish reaches or is beyond the outer circle. Note that fish usually turns around before reaching complete darkness. (C) Larval zebrafish prefer light over real shadows (mostly temporal darkening). Upper left panel: the light source comes from below the arena, and a ring-shaped piece of material that blocks visible light (but transmits infrared light) is positioned under the arena, i.e., a strong shadow is created for the ring-shaped area. Lower left panel: trajectory of a fish over a session of 6 min. The fish mainly stays within the center circle of the dish where they can see the light source; when it crosses the border into the shadow area it quickly returns back to the circle. Insets show 3 trajectory segments (rotated into same orientation), direction indicated by the red, green and blue arrowhead, yellow dots mark the points where fish enters the shadow (crossing the border that is marked by dashed red line). Note similarity to [file Presentation1.ZIP › 81220_Engert_Supplementary Figure_4.TIF]
